# Overlap of expression Quantitative Trait Loci (eQTL) in human brain and blood

**DOI:** 10.1186/1755-8794-7-31

**Published:** 2014-06-03

**Authors:** Marna McKenzie, Anjali K Henders, Anthony Caracella, Naomi R Wray, Joseph E Powell

**Affiliations:** 1 The University of Queensland, Queensland Brain Institute, Brisbane, Queensland, Australia; 2 Queensland Institute of Medical Research, Brisbane, Herston, Australia

## Abstract

**Background:**

Expression quantitative trait loci (eQTL) are genomic regions regulating RNA transcript expression levels. Genome-wide Association Studies (GWAS) have identified many variants, often in non-coding regions, with unknown functions and eQTL provide a possible mechanism by which these variants may influence observable phenotypes. Limited access and availability of tissues such as brain has led to the use of blood as a substitute for eQTL analyses.

**Methods:**

Here, we evaluate the overlap of eQTL reported in published studies conducted in blood and brain tissues to assess the utility of blood as an alternative to brain tissue in the study of neurological and psychiatric conditions. Expression QTL results from eight published brain studies were compared to blood eQTL identified in from a meta-analysis involving 5,311 individuals. We accounted for differences in SNP platforms and study design by using SNP proxies in high linkage disequilibrium with reported eQTL. The degree of overlap between studies was calculated by ascertaining if an eQTL identified in one study was also identified in the other study.

**Results:**

The percentage of eQTL overlapping for brain and blood expression after adjusting for differences in sample size ranged from 13 - 23% (mean 19.2%). Amongst pairs of brain studies eQTL overlap ranged from 0 - 35%, with higher degrees of overlap found for studies using expression data collected from the same brain region.

**Conclusion:**

Our results suggest that whenever possible tissue specific to the pathophysiology of the disease being studied should be used for transcription analysis.

## Background

The combination of expression profiling and genotyping from microarrays has led to estimation of correlations between genetic variants such as Single Nucleotide Polymorphisms (SNPs) and RNA transcript expression levels [1-3]. Expression quantitative trait loci (eQTL) are the genomic loci that influence RNA transcript expression levels. The contribution of eQTL to underlying variation in complex traits such as disease susceptibility has been studied extensively since expression levels were first described as quantitative traits with a genetic basis. Gene expression is the primary detectable phenotype in the development of complex traits; therefore expression levels act as an intermediate phenotype between genetic architecture and observable multifactorial traits such as common diseases. It has been shown that SNPs associated with common diseases identified by genome-wide association studies (GWAS) are enriched for expression-affecting SNPs (eSNPs) [4,5]. Furthermore, most associated variants are not located in protein coding regions and are instead highly enriched for regulatory regions of the genome [6], suggesting that for many variants, the functional mechanism by which they affect disease susceptibility is through gene regulation. The majority of eQTL that have currently been identified are located within the *cis*-region (typically +/− 1 MB) of the transcription start site (TSS). Expression-QTL located outside of the *cis*-region are termed *trans*-eQTL and likely reflect an indirect relationship of the SNP on gene expression. *Cis*-eQTL tend to have larger effect sizes [6], are more often replicated across multiple studies [7] and more likely to reflect a direct functional relationship between the SNP and the measured expression levels.

The pathophysiology of most common diseases is restricted to a limited number of tissue types or organ systems. Therefore, to understand the mechanisms of disease susceptibility and develop preventative and targeted therapies, we ultimately require knowledge of genetic control of regulatory variation in many different tissues. A common limitation in disease genomics studies is availability of pathologically relevant tissue on which to measure expression. Most studies instead rely on inferences drawn from readily available (‘proxy’) tissues, typically whole blood. However, the genetic control of RNA transcription is known to vary between tissues [5,8-11], and the phenotypic correlations and co-expression can be low [12]. Expression-SNPs that are also associated with disease susceptibility are more likely to affect expression levels in a tissue-specific manner compared to eSNPs with no known disease association [5]. Due to mainly study-design limitations most eQTL analyses focus on gene expression measured in a single tissue type [9,13-18], posing limitations when attempting to study more inaccessible tissues, such as the brain.

Using expression levels measured from brain tissue for eQTL analysis poses several unique problems. First, the brain is a collection of numerous cell types meaning that gene expression levels are not consistent throughout the entire structure [7,19]. This introduces cell type specificity within the brain itself. Secondly, collection of tissue samples in post-mortem requires consideration of several novel factors not relevant when examining other tissue types – such as cause of death, post-mortem RNA integrity and post-mortem interval [20]. This has led to the search for alternate methods for evaluation of eQTL within the brain.

One approach is to use expression levels measured from more accessible tissues, such as blood, as a substitute to gain insight into gene regulation underlying brain-related traits such as psychiatric and neurological disorders [21-25]. When such approaches are used an important question is: To what degree is the genetic control for expression the same in brain and blood tissues? Difficulties in the collection of expression data from both blood and brain tissue for the same individuals mean methods are required to assess the overlap of genetic control. Using results from published studies represents a practical solution. Here we report a comprehensive evaluation of the degree of eQTL overlap between blood and brain and aim to determine the validity of using blood as a surrogate for brain tissue. We have sought to account for differences in expression and genotype platform arrays as well as to correct for differences in sample size and thus differences in statistical power between studies.

## Methods

### eQTL results

Published eQTL results and supporting information were obtained from eight eQTL studies carried out on expression levels measured in the brain using high-throughput arrays [7,10,26-31]. Full details of each study are given in Additional file 1: Table S1. Each of these studies evaluated the association between transcript probe expression levels and SNP genotype using linear regression models. Significant eQTL were determined at a *p*-value threshold that was specific to the study. Studies used different metrics and protocols to identify eQTL and so we sought to standardize results between studies by producing lists of eQTL and eSNPs that reached the study-wide significance level of *α*  =  0.05. The number of significant eQTL reported ranged from 52 to 2,975, which represents between <1 - 19% of the probes analyzed in each study. This range most likely reflects differences in sample size, protocols and statistical analyses between the studies. Three studies [10,26,30] reported *cis* associations only, while the remaining five reported both *cis* and *trans* associations (*trans* eQTL are those located outside of the *cis*-region definition of the study), including SNPs distal to the associated transcript [7,27-29,31]. Details of the significant eQTL and eSNPs reported in each study are given in Table 1.

**Table 1:** Summary of each study included in the comparison

**Study**	**Sample Size**	**Tissue(s)**	**Number of genes with an eQTL**		**Percentage of probes with an eQTL**	**Total number of probes**
Westra *et al*. [ 32]	5,311	Peripheral blood	4,909		33	14,586
Colantuoni *et al*. [26]	269	Prefrontal Cortex	455		2	30,176
Gibbs *et al*. [7]	150	Caudal Pons	997	278	5	22,184
		Cerebellum		318		
		Frontal Cortex		331		
		Temporal Cortex		385		
Heinzen *et al*. [10]	93	Frontal Cortex	52		< 1	~22,000
Kim *et al*. [27]	165	Cerebellum, Frontal Cortex, Thalamus, Temporal Cortex	648	211	*	
		Hippocampus, Frontal Cortex		594		
Liu *et al*. [28]	127	Prefrontal Cortex	1,063		15	
Myers *et al*. [31]	193	Cortex (Pooled data from 20% frontal, 70% temporal and 1% parietal)	2,975		19	
Webster *et al*. [29]	364	Cortex (Pooled from 21% frontal, 73% temporal, 2% parietal and 3% cerebellar)	743		9	8,650
		Cortex (Pooled from 18% frontal, 60% temporal, 10% parietal and 13% cerebellar)				
Zou *et al*. [30]	374	Cerebellum	686		3	24,526

*Exact number of transcripts tested is not given.

All brain tissue samples were collected post-mortem. Some studies reported eQTL for expression collected in multiple brain regions or from samples with different neuropathologies. Where possible, eQTL overlap was evaluated for separate tissues and neuropathologies, however, most studies pooled results from different tissues or from individuals with different neurological/psychiatric conditions. Therefore, separate analysis was not always possible. For example, two of the studies included samples from individuals diagnosed with schizophrenia, bipolar disorder, or major depressive disorder [27,28], whilst others pooled samples with pathologies such as Alzheimer’s disease and progressive supranuclear palsy [29,30]. Only four studies used expression data collected from a single region of the brain [10,26,28,30], whilst the remainder, with the exception of Gibbs *et al*. [7], combined data acquired from several brain regions.

The eQTL results from the eight brain studies were compared to results from a large meta-analysis of eQTL in for expression levels measured in whole blood [32]. The Westra *et al*. analysis is the largest eQTL study published to date and comprises of a meta-analysis of 5,311 individuals in a discovery phase and 2,775 individuals in a replication phase. All expression levels were measured in peripheral blood and corrected for batch effects and cell counts. SNP effects were estimated using a weighted Z-score of the beta values calculated using a linear regression model in each study cohort. Significance was determined using permutations and a study-wide threshold of false discovery rate (FDR) of 0.05. Cis-eQTL were identified for a total of 4,909 genes (33% of all tested) and trans-eQTL for 430 genes (3%).

This study used previously published data. The research was approved by the University of Queensland Human Ethics Review Board and the QIMR Berghofer Medical Research Institute Institutional Review Board for Research on Human Subjects.

### eQTL overlap

The eQTL studies included in this analysis used a variety of high-throughput expression arrays (Additional file 1: Table S1). We sought to overcome the degree of probe overlap between by comparing eQTL for probes that tag the same gene exon. We evaluated the overlap of eQTL identified in blood [32] and each of the brain studies as well as between each pair of brain studies. The latter allows us to draw conclusions on eQTL overlap for different brain regions or tissues as well as potential impact of psychiatric or neurological disorders. The following procedures were followed to ascertain the overlap of eQTL between peripheral blood and each brain study and between each pair-wise comparison of brain studies.

The list of significant (study-wide *α* = 0.05 eQTL for studies *i* and *j* were first assessed for probes containing an eQTL in both studies. For each eQTL present in studies *i* and *j*, overlapping eQTL were determined using two approaches. Firstly, SNPs with the smallest *p*-value (eSNPs) from eQTL were compared based on their recorded presence in both studies. This provides a basic comparison of all overlapping eQTL that can be identified from standard reported results. However, the mean overlap between *i* and *j* is expected to be biased downwards due to differences in SNP array platforms and inconsistencies in linkage disequilibrium (LD) between study populations. We accounted for this by applying a second approach that used known SNP proxies [33] from the 1000 Genomes study (CEU panel). SNP proxies were defined as SNPs in high LD (*r*^*2*^ > 0.8) and within 100 kb of the original genotyped eSNPs. A list of SNP proxies for each eSNP was generated for all studies. The SNP proxies for eQTL in studies *i* and *j* were then compared to find overlapping proxy eQTL (hereon termed eQTL_proxy_). This approach provides a realistic estimate of the true eQTL overlap that would be expected if all studies had used the same genotyping platform.

The power to detect eQTL is partly a function of sample size. Thus, comparison of eQTL between two studies of different sample sizes is expected to downwardly bias the estimate of the true proportion overlapping due to the lower power of the smaller study. We addressed this problem by using a method presented by Ding *et al*. [32] that adjusts for the difference in samples sizes, and thus statistical power, to re-estimate eQTL overlap expected under the scenario of equal sample size [34]. Following the notation of Ding *et al*., the estimate of power-adjusted overlap, we start with the lists of eQTL identified in studies *i* and *j*. EQTL are determined based on significance threshold*,* α, which corresponds to a false discovery rate (FDR) of 0.05. From these lists we determined the observed percentage of eQTL overlapping between studies (*π*_*raw*_). Because both eQTL lists are inevitably incomplete, *π*_*raw*_ will be an underestimate of the true level of overlap, *π*. If study *j* is of smaller sample size than *i*, then the power to detect ‘true’ overlapping eQTL will be lower. The power-adjusted expected overlap in significant eQTL (${\hat{\pi }}_{\mathrm{adjusted}}$) is calculated as [32]

(1) $${\hat{\pi }}_{\mathrm{adjusted}}=\frac{{\hat{\pi }}_{\mathrm{raw}}-\alpha_{j}}{\left(1-\mathrm{FD}R_{i}\right)\left(\mathrm{powe}r_{j}-\alpha_{j}\right)}$$

where *power*_*j*_ is determined from the effect sizes of the overlapping eQTL, the sample size and *a*_*j*_,

(2) $$\mathrm{po}\hat{w}er_{j}=\frac{\mathrm{po}\hat{w}er_{j\_\mathrm{raw}}-\mathrm{FD}R_{i}*a_{j}}{1-\mathrm{FD}R_{i}}$$

*power*_*j_raw*_ is defined as the statistical power of study *i* with a sample size matching study *j* to detect all identified eQTL from study *i*. Since the blood-based eQTL analysis was the largest study by far, we used this sample to estimate *power*_*j_raw*_ by:

1. From the Westra *et al*. list of eQTL we sampled two mutually exclusive and independent datasets (*i*_*1*_ and *i*_*2*_), with the sample size of both *i*_*1*_ and *i*_*2*_ equal to that of study *j*.

2. EQTL were identified in datasets *i*_*1*_ and *i*_*2*_, assuming significance threshold of study-wide FDR of 0.05.

3. *power*_*j_raw*_ was calculated as the proportion of eQTL identified in *i*_*1*_ that are also significant in *i*_*2*_.

4. ${\hat{\pi }}_{\mathrm{adjusted}}$ was calculated (equations 1 and 2) between each brain study and the Westra *et al.* blood eQTL study.

### Functional characterization of overlapping eQTL

A KEGG pathway enrichment analysis was used to assess shared biological functionality amongst genes with either overlapping or independent eQTL. From each pairwise comparison of blood and brain eQTL, genes listed as having either overlapping or non-overlapping eQTL were analyzed for shared functionality using DAVID [35]. Significance of pathway enrichment was determined from a modified Fisher’s test, which represents the probability that a set of genes of related terms are presented at a given percentage in the list. Multiple testing was accounted for using a Benjamini-Hochberg FDR of 0.05 [36].

## Results

### Blood and brain eQTL overlap

For *cis*-acting eQTL only, the overlap of eQTL between blood and brain studies ranged 0.1–14.2% (mean 4.3%) (Table 2). In total 4,909 *cis*-eQTL were found for blood gene expression, of which 156 were also identified for cerebellum and temporal cortex expression [29]. Overlapping *trans*-eQTL were identified from 5 studies, but the number of genes was low (2–35) (Table 2). *Trans*-eQTL typically have smaller effect sizes than *cis*-eQTL and suffer from a greater multiple testing burden, resulting in lower power for detection compared to *cis*-effects. These estimates of overlap are likely to be an under-estimate of the true level of overlap as the ‘replication’ studies in brain tissue have smaller sample sizes and thus lower power than Westra *et al*. [32]. Indeed, if we use the brain studies as the ‘discovery’ and Westra *et al*. as ‘replication’ the proportion of *cis*-eQTL overlap ranges 5.7-70% (mean 22%). These results are from analyses where the probes are matched to gene exons and using SNP proxies of eSNPs. Verification of the robustness of our results to deviation in the SNP proxy LD threshold is shown in Additional file 2: Table S2. We believe our results provide an estimate of the proportion of overlap that is as accurate as possible given the limitations of the published data. Using the method published by Ding *et al*. [34] we attempted to account for differences in the sample sizes between the blood and brain studies (see Methods). Accounting for the smaller sample sizes of brain studies, the expected proportion of blood eQTL that would be identified had the brain studies be of equal size (*n* = 862), ${\hat{\pi }}_{\mathrm{adjusted}}$, ranges 13% -23% (mean = 19.2%) (Table 3), suggesting that observed estimates (*π*_*raw*_) are under-estimates due to lower sample sizes in brain studies. It is important to note that the method used to estimate the adjusted overlap assumes that the distributions of eQTL effect sizes are similar for overlapping and non-overlapping eQTL [34]. There is evidence to suggest this assumption may be incorrect and the implications of this are discussed below.

**Table 2:** Summary of blood eQTL overlap with each brain eQTL study

**Study**	**Number of overlapping eQTL**	**Percentage of genes in blood with overlapping eQTL in brain study**	**Percentage of genes in brain study with overlapping eQTL in blood**	**Mean number of overlapping SNPs per gene**	**Mean R**^**2 **^**(blood)**^**1**^	**Mean R**^**2 **^**(brain study)**^**1**^	**Mean distance (kb) of overlapping SNP from probe**^**1**^
**CIS**							
Colantuoni *et al*. [17]	94 (12)	1.9 (0.2)	20.1 (2)	5.5	0.14	Data unavailable	18.6
Gibbs *et al*. [12]	697 (34)	14.2 (0.6)	70 (3.4)	15.7	0.19	0.32	62.8
Heinzen *et al*. [7]	7 (0)	0.15 (0)	13 (0)	11.1	0.11	0.14	48.1
Kim *et al*. [18]	66 (8)	1.3 (0.1)	10 (1.2)	7.6	0.15	Data unavailable	39.7
Liu *et al*. [19]	59 (7)	1.2 (0.1)	5.7 (0.8)	4.8	0.10	0.15	25.1
Myers *et al*. [22]	507 (49)	10.3 (1)	17 (1.7)	7.1	0.13	Data unavailable	18.5
Webster *et al*. [20]	133 (17)	2.7 (0.3)	18 (2.2)	3.4	0.12	0.19 AD	69.0
						0.15 Controls	
Zou *et al*. [21]	156 (16)	3.1 (0.3)	23 (2.3)	5.6	0.13	0.22^3^	26.1^2^
**CIS AND TRANS**							
Gibbs *et al*. [12]	712 (38)	14.3 (0.6)	0.71 (3.4)	15.8	0.18	0.32	
Kim *et al*. [18]	69 (8)	1.4 (0.1)	10.6 (1.2)	7.8	0.14	Data unavailable	
Liu *et al*. [19]	61 (7)	1.2 (0.1)	5.7 (0.8)	4.3	0.11	0.15	
Myers *et al*. [22]	542 (54)	10.3 (1)	18 (1.7)	7.6	0.14	Data unavailable	
Webster *et al*. [20]	142 (19)	2.7 (0.3)	19 (2.2)	3.1	0.14	0.18 AD	
						0.13 Controls	

^1^Only for identical SNP:Gene associations.

^2^Cis = ± 100 kb.

^3^Only 139/514 (27.0%) of SNP:Gene associations had R^2^ data.

R^2^ is the proportion of transcript level variance explained by the overlapping SNP.

*AD* – Late-onset Alzheimer’s disease.

Numbers shown in brackets indicate contribution of proxy SNPs to total value. The numbers in parentheses are those found using proxy SNPs.

**Table 3:** Estimation of the expected degree of overlap between blood eQTL and each of the brain studies should the sample sizes be equal

**Study**	$\mathrm{po}\hat{w}er_{j\_\mathrm{raw}}$	${\hat{\pi }}_{\mathrm{adjusted}}$	${\hat{\pi }}_{\mathrm{raw}}$
Colantuoni *et al*. [17]	0.42	19%	5%
Gibbs *et al*. [12]	0.59	21%	7%
Heinzen *et al*. [7]	0.19	13%	0.5%
Kim *et al*. [18]	0.88	19%	9%
Liu *et al*. [19]	0.67	20%	6%
Myers *et al*. [22]	0.63	22%	6%
Webster *et al*. [20]	0.57	17%	4%
Zou *et al*. [21]	0.84	23%	11%

### Functional characterization

To investigate if both overlapping and non-overlapping eQTL have a shared functionality we performed a pathway analysis using DAVID [35]. The lists of overlapping and non-overlapping eQTL genes are given in Additional file 3: Table S4. For both sets of genes with overlapping and non-overlapping eQTL no statistically significant enrichment was found for functional annotation or Gene Ontology (GO) terms. This suggests that the genes with overlapping eQTL have similar functional roles in brain and blood cell types and tissues. Amongst the eight comparisons, 268 independent genes were found to have overlapping cis-eQTL with blood. Of these 55 (21%) had an eQTL observed in more than two brain studies. The genes that most consistently reported an overlapping eQTL were *NSFL1C* and *PEX6* (reported in six of the brain eQTL studies), and *CDK5RAP2, CDS2, CHURC1, CRIPT, HMBOX1, MRPL43, NAPRT1, NSUN2, RABEP1, ZNF266, ABHD12,* and *PILRB* (reported in five of the brain eQTL studies) (Additional file 4: Table S3).

### Brain region eQTL overlap

The brain studies examined here report eQTL for expression levels measured in cells collected from several different tissues and brain regions. Given specific differences in the aetiology of many neurological disorders, one important consideration is whether eQTL overlap between different brain regions. To investigate this we analyzed overlap using our observed and SNP proxy methods for each pairwise combination of brain studies. *Cis*-eQTL overlap is shown in Table 4, while the only observable *trans*-eQTL overlap was in Myers *et al*. [31] and Webster *et al*. [29] at 0.2% and 1.5% respectively (including SNP proxies). The variability in overlap between studies remains high even when comparing the eQTL data generated from the same brain region. For example, in the two studies examining the prefrontal cortex, the proportion of overlapping genes with a significant eQTL was only 1% to 7% (Table 4) [26,28]. However, the proportion of overlapping genes with a significant *cis*-eQTL from cerebellar tissues in Gibbs *et al*. [7] and Zou *et al*. [30] was much greater. Of the genes from Gibbs *et al*. [7] with a significant *cis*-eQTL in the cerebellum, 39% were also observed in Zou *et al*. [30]. Conversely, 8% of the genes with a significant *cis*-QTL from cerebellar tissue in Zou *et al*. [30] were reported in Gibbs *et al*. [7].

**Table 4:** Overlap of eQTL from each of the pairwise comparisons of brain studies

**Replication dataset**		**Discovery dataset**															
		**Colantuoni**	**(P)**	**Gibbs**	**(P)**	**Heinzen**	**(P)**	**Kim**	**(P)**	**Liu**	**(P)**	**Myers**	**(P)**	**Webster**	**(P)**	**Zou**	**(P)**
	**Colantuoni**	/	/	16.0	0.7	13.6	0	14.1	11.3	0.6	0.6	7.4	3.7	6.1	3.6	10.4	0.6
	**Gibbs**	9.9	0.4	/	/	0	0	9.5	1.2	0.6	0.1	7.4	0.6	15	0.7	10.6	1
	**Heinzen**	0.7	0	0	0	/	/	0.3	0.3	0	0	0	0.	0	0	0	0
	**Kim**	20	16.1	22	2.8	9.1	9.1	/	/	2.3	0.7	14.7	3.4	16.1	1.4	11.8	9.5
	**Liu**	6.8	6.4	10.7	1.1	0	0	18.9	5.9	/	/	6.6	1.9	8.6	1.1	4.2	3.5
	**Myers**	10.6	5.3	17.1	1.4	0	0	14.7	3.4	0.8	0.2	/	/	35.4	0	9.6	4.2
	**Webster**	3.7	2.2	14.9	0.7	0	0	6.9	0.6	0.5	0.1	15.3	0	/	/	3.5	1.5
	**Zou**	15.6	0.9	26	2.5	9.1	0	12.5	10.	0.6	0.5	10.2	4.5	8.6	3.6	/	/

The percentage of genes from the discovery dataset that were found to have an overlapping eQTL in the replication dataset is shown. The percentage listed under (P) indicates the contribution of proxy SNPs to the total overlap reported.

**Figure 1:**
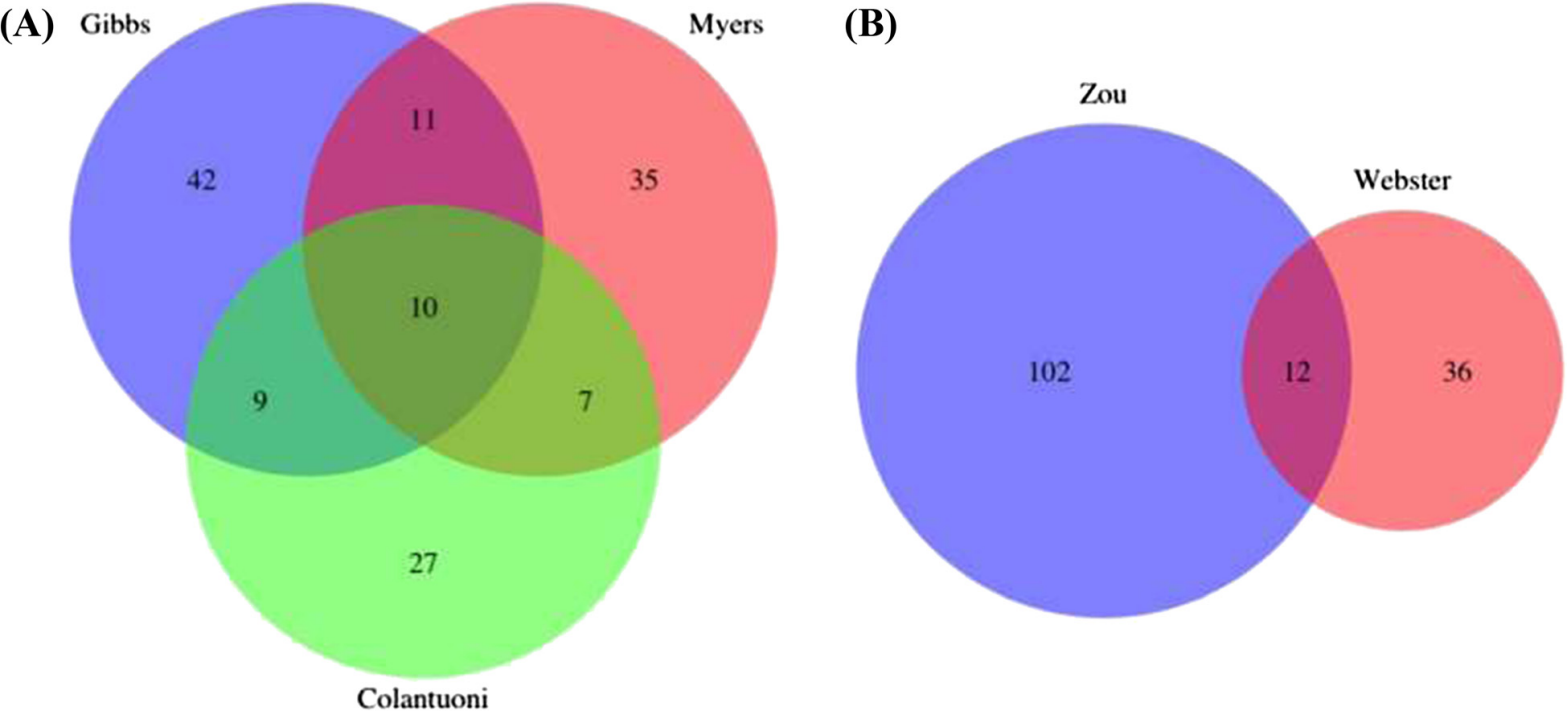
**Venn diagram showing the overlap in genes with common eQTL between brain studies.** In figure **(A)** all studies used samples with healthy or normal neuropathology, and tissue samples were collected from different cortical brain regions; Cortex (Myers), Prefrontal Cortex (Colantuoni) and Temporal Cortex (Gibbs). In **(B)** samples are collected from the Temporal Cortex and Cerebellum in individuals with normal or healthy neuropathology (Zou) and Cortex in individuals with late onset Alzheimer’s disease (Webster).

To evaluate eQTL overlap between different brain regions we calculated the average overlap for studies that used expression levels measured from the same region compared to the average for studies using expression measured in different regions. For each comparison we chose the dataset with the largest sample size as the ‘discovery’ sample (Table 2). The mean overlap between studies with the same tissue is 20% and for studies using different tissues is 12%. Following this, we chose pairs of studies that both collected samples from healthy or normal neuropathology and compared those to studies using healthy versus diseased patients. The mean overlap between healthy-healthy studies was 19% and between healthy-disease was 13% (Figure 1).

Of the 765 genes found to have a *cis*-eQTL in one of the two studies involving cerebellar tissue [7,30], 137 (18%) were also reported to have a *cis*-eQTL in blood. This proportion increased considerably when evaluating eQTL reported by both cerebellar studies – 25 of the 68 (37%) genes reported in both cerebellar studies also displayed a significant *cis*-eQTL association in blood.

Through contrasting distinctive neuropathologies, Liu *et al*. [28] showed that different psychiatric conditions had little effect on eQTL mapping compared to healthy controls. Consequently, most studies included here had pooled eQTL results from cohorts where samples have differing psychiatric conditions [27,28]. However, Webster *et al*. [29], reported eQTL that had a significant effect only amongst individuals with late-onset Alzheimer’s. The overlap of these eQTL with those found in blood is summarized in Table 5. Notably, blood eQTL overlap with Alzheimer’s disease interacting eQTL was three times lower than eQTL without a significant interaction with disease status.

**Table 5:** **Summary of Westra et al.**[32]**blood eQTL overlap with eQTL from Webster *et al*.**[29]**which were found to have an interaction with late-onset Alzheimer’s disease (AD) status as well as those independent of disease status**

**Effect of diagnosis**	**Number of overlapping eQTL**	**Percentage of genes in blood with overlapping eQTL in Webster et al.**	**Percentage of genes in Webster et al. with overlapping eQTL in blood**	**Mean number of overlapping SNPs per gene**	**Mean *R***^***2 ***^**(blood)**^**1**^	**Mean *R***^***2 ***^**(Webster *et al*.)**^**1**^	**Mean distance (kb) of overlapping eSNP from TSS**^**1**^
AD interaction	67 (1)	1.3 (0)	9 (0.1)	1.4	0.12	0.20 Cases	78.4
						0.09 Controls	
No interaction	111 (14)	2.2 (1)	15 (1.8)	4.0	0.12	0.15 Cases	52.9
						0.13 Controls	

^1^Only for identical SNP:Gene associations.

Numbers shown in brackets indicate contribution of proxy SNPs to total value. R^2^ is the proportion of transcript level variance explained by the overlapping SNP.

## Discussion

We have quantified the overlap in eQTL identified from eight brain studies with those found in blood. A key motivation of this work was to provide information on the utility of using blood as a surrogate for brain tissue when trying to understand the role of gene expression in neurological and psychiatric conditions. Previous work had shown that the transcriptome organization is poorly preserved between brain and blood, although the conservation was stronger for certain hub genes [12].

If we solely consider the overlap amongst the eight brain studies, there is relatively low, but highly significant, levels of replication (p = 1.4e^−8^). The greatest degree of overlap (35%) of *cis*-eQTL is between two studies that focused on cortex regions [28,30]. If *trans*-eQTL are included in our analysis then the percentage overlap falls to 15%. The low level of overlap is likely due to a number of contributing factors. Firstly, we are comparing individuals from different population demographics whose expression levels have been measured using different laboratory procedures and protocols. Difference in expression profiles as a result of these variables is well documented [26,37,38]. Secondly, individuals from different disease cohorts were included together in the analyses. The effect of disease status on eQTL is still unclear; Liu *et al*. [28] found that psychiatric disease status had minimal effects on eQTL mapping, whilst Webster *et al*. [29] showed that most of the Alzheimer’s disease associated eQTL effects were not found in healthy controls. Thirdly, the studies sampled and often combined data from different regions of the brain. Yet it has been shown that expression profiles generated from different brain regions are distinct [7,19]. Additional and larger studies are needed to clarify and quantify the true overlap.

Where possible we used approaches to account for differences in microarray and genotype platforms that could contribute to a low concordance in eQTL overlap between studies. EQTL and eSNP were matched for expression probes located within the same exon and SNP proxies were generated and analyzed for all eSNPs. The proxy SNPs included all known common SNPs within 100 kb region that are linked to the eQTL by LD. Therefore, if the eQTL was present in each study, it should be detected using this method.

Brain eQTL studies, with smaller sample sizes, lack statistical power compared to the blood meta-analysis [32]. To address this, we employed an approach [32] which re-estimates the expected degree of eQTL overlap, should the sample size of the brain study match that of Westra *et al*. Although it has been estimated that a sample size of 100 individuals is sufficient for 80% power in eQTL studies [39], the power to detect an effect across multiple studies is reduced when an eSNP has a small effect size or when multiple loci are controlling transcript expression levels.

The highest proportion of *cis*-eQTL overlap between blood and any brain study was 14.2% [7]. Under a null hypothesis of no eQTL overlap, these observed values are much greater than would be expected by chance (*p* = 2.7e^−11^). We can calculate the expected amount of overlap under the null hypothesis that there is no true overlap using the proportion of probes with identified eQTL in each study. For example, the amount of overlap expected between Westra *et al*. [32] and Gibbs *et al*. [7] based on chance would be equal to the percentage of probes analyzed in Westra *et al*. that were found to have an eQTL (33%), multiplied by the percentage of probes examined in Gibbs *et al*. [7] that were found to have an eQTL (5%), which is 1.6%.

Consistent with findings from other tissues, the genetic regulation of transcript expression levels within the brain and blood appears to be largely tissue specific [8,10]. Our results presented here use data collected among different individuals and thus will underestimate the true overlap because of inconsistencies in study design, environmental effects and allele frequencies of SNPs. Studies such as GTEX [40], which is collecting samples from multiple tissues in the same individuals, can be used to provide a more complete understanding of identifiable eQTL overlap between these tissues. Importantly, it would also allow an accurate quantification of the direction of allelic effects between tissues. Here, due to unavailability of beta estimates, we have assumed the direction of eQTL effects between tissues is the same. As is expected, the overlap between brain and blood was consistently lower than the amount of overlap among brain eQTL studies. This provides support for the methods used for eQTL comparison in this study. The high level of overlap identified between studies using samples with disparate medical history provides support for the hypothesis that neurological conditions have little impact on eQTL effect estimates [28].

Table 2 provides information on the genes with an eQTL in both brain and blood, presented as a proportion of eQTL identified in the studies. The proportion of eQTL overlap was on average greater when the brain eQTL study was used as the ‘discovery’ cohort. This is likely due to the larger sample size of the blood eQTL study.

The mean genomic distance of overlapping blood–brain *cis*-eSNP from the probe TSS was 38.5 kb (not including proxy SNPs) – substantially less than the 121 kb average distance for brain eQTL reported by Gibbs *et al*. [7]. This is in agreement with previous findings, which show that eQTL found in multiple tissues tended to localize closer to the TSS than tissue-specific eQTL [9]. It is also known that eQTL found in multiple tissues have larger effect sizes than average, which could lead to a upwards bias in the reported percentage of overlapping eQTL between brain and blood [5].

The pathophysiology of many neurological and psychiatric conditions is often localized to specific brain regions. For example, prefrontal and temporal cortex abnormalities have been repeatedly associated with schizophrenia [41-43] while affective disorders such as bipolar disorder and major depressive disorder have been linked with dysfunction in several brain regions – cingulate cortex, amygdala, thalamus, hippocampus and the frontal lobe [44-48]. Similarly, neurological disorders tend to act within particular brain structures – degeneration in the substantia nigra is a hallmark of Parkinson’s disease [49] and one of the primary characteristics of Alzheimer’s disease is the prevalence of neurofibrillary tangles and amyloid plaques, particularly in medial temporal lobe structures [50,51]. Thus, analysis of regions specifically affected by the disorder of interest is likely to be more relevant when attempting to understand the contribution of eQTL to disease susceptibility. Evaluating brain-region specific overlap among the brain studies demonstrated highly variable results. Amongst the brain study comparisons there was a higher concordance of eQTL overlap when comparing results from expression levels measured in the same brain region, notably cerebellar tissue.

To consider the effects of neuropathologies on eQTL overlap between brain and blood, individuals with several neurological and psychiatric conditions were included in our comparisons. As most studies pooled data from healthy and diseased individuals, a separate analysis of blood eQTL overlap was not always possible. The exception is Webster *et al*. [29], who reported eQTL with a significant effect only amongst individuals with Alzheimer’s disease diagnosis. The overlap of blood eQTL with Alzheimer’s disease associated eQTL was lower than eQTL that showed no disease specific effects, suggesting the possibility of increased tissue specificity of genetic regulation of expression levels in individuals with Alzheimer’s disease.

## Conclusion

There are several recognizable limitations in this study such as demographic differences, disease status, brain cell heterogeneity, sample size and potential differences in protocols and array platforms. Where possible we have used methods to address these limitations, leading to a picture of eQTL overlap that represents the best-case scenario should studies have used the same array platforms and equal sample sizes. Ideally, measuring expression levels for multiple brain regions and blood in the same individuals would provide the best approach to more fully evaluate the overlap. In summary, although the genetic regulation of expression levels appears to act in a primarily tissue-dependent manner, overlap is still observed although there appears to be no functional differences in the genes with overlapping eQTL. Our results suggest that whenever possible tissue specific to the pathophysiology of the disease being studies should be used for transcription analysis. However, given the availability of blood, and the likely increases in sample size, analysis should not be deemed worthless for informing on brain eQTL associated with neurological and psychiatric conditions.

## Competing interests

The authors declare that they have no competing interests.

## Authors’ contributions

JEP and NRW designed the study. MM and JEP performed analyses and drafted the manuscript. AKH and AC performed experiments to generate data. All authors read and approved the final manuscript.

## Pre-publication history

The pre-publication history for this paper can be accessed here:

http://www.biomedcentral.com/1755-8794/7/31/prepub

## Supplementary Material

Additional file 1: Table S1 Complete summary of each study included in comparison. Click here for file

Additional file 2: Table S2 Overlap of eQTL was evaluated using the SNP proxy lists generated under two linkage disequilibrium threshold (*r*^*2*^ = 0.8 and *r*^*2*^ = 0.5). Click here for file

Additional file 3: Table S4 Gene lists with overlapping eQTL found for whole blood. Overlapping eQTL genes are given separately for each brain study. Click here for file

Additional file 4: Table S3 Genes with an overlapping eQTL reported in Westra et al. and brain studies. Click here for file
