# Supplementary material for: Overlap of expression Quantitative Trait Loci (eQTL) in human brain and blood
Source: BMC Med Genomics. 2014 Jun 3;7:31. doi: 10.1186/1755-8794-7-31 (PMC4066287; doi:10.1186/1755-8794-7-31)
Supplement: Additional file 1: Table S1 — Complete summary of each study included in comparison. [file 1755-8794-7-31-S1.docx]

**Supplementary Table 1 |** Complete summary of each study included in comparison

| **Study** | **# of Individuals** | **Tissue** | **# of Tissue Samples** | **Neuropathology** | **# of eSNPs** | | **# of genes with an eQTL at p<0.05** | | **Age** | **Gender** | **Ethnicity** | **Expression Array Platform** | **SNP Array Platform** | **Cis only or cis/**  **trans** |
| --- | --- | --- | --- | --- | --- | --- | --- | --- | --- | --- | --- | --- | --- | --- |
| Westra *et al*. [ [32](#_ENREF_32)] | 5,311 | Peripheral blood | 5,311 | Normal | 79,412 | | 4,909 | | 14-82 | 50% male  50% female | European Caucasian | Illumina HumanHT-12 v4.0 and v3.0 | Illumina 610-Quad | Cis and trans |
| [Colantuoni *et al*. [26](#_ENREF_26)] | 269 | Prefrontal Cortex | 269 | Normal | 1 628 | | 455 | | Fetal - 80 mean 27.8 | 66% male  34% female | 147 African-American  112 Caucasian  6 Hispanic  4 Asian | Illumina Human 49K Oligo array | Illumina Infinium II 650K  or  Illumina Infinium HD Gemini 1M Duo | Cis only |
| [Gibbs *et al*. [7](#_ENREF_7)] | 150 | Caudal Pons | 142 | Normal | 19 509 | 3 415 | 997 | 278 | 15-101  mean 46.2 | 69% male  31% female | Caucasian | Illumina Human  Ref-8 Expression | Illumina Infinium Human  Hap550 | Cis and trans |
|  |  | Cerebellum | 143 |  |  | 5 244 |  | 318 |  |  |  |  |  |  |
|  |  | Frontal Cortex | 143 |  |  | 5 515 |  | 331 |  |  |  |  |  |  |
|  |  | Temporal Cortex | 144 |  |  | 5 335 |  | 385 |  |  |  |  |  |  |
| [Heinzen *et al*. [10](#_ENREF_10)] | 93 | Frontal Cortex | 93 | Normal | 52 | | 22 | | 34-90  mean 74 | 59% male  41% female | Caucasian | Affymetrix Huamn ST 1.0 | Illumina Human Hap550K | Cis only |
| [Kim *et al*. [27](#_ENREF_27)] | 165 | Cerebellum,  Frontal Cortex,  Thalamus,  Temporal Cortex | 60 | 15 Schizophrenia | 1 046 | 305 | 648 | 211 | 25-62 mean 44.2 | 60% male  40% female | 12 Caucasian  3 Asian | Affymetrix  HGU133A  or  Affymetrix HGU1332.0+  or  Affymetrix HGU95Av2 | Affymetrix Human SNP Array 5.0 | Cis and trans |
|  |  |  |  | 15 Bipolar |  |  |  |  | 25-61 mean 42.3 | 60% male  40% female | 14 Caucasian  1 African American |  |  |  |
|  |  |  |  | 15 Major Depression |  |  |  |  | 30-65 mean  46.4 | 60% male  40% female | 15 Caucasian |  |  |  |
|  |  |  |  | 15 Normal |  |  |  |  | 29-68 mean 48.1 | 60% male  40% female | 14 Caucasian  1 African American |  |  |  |
|  |  | Hippocampus,  Frontal Cortex | 105 | 35 Normal |  | 741 |  | 594 | 31-59  mean 44.1 | 74% male  26% female | 35 Caucasian |  |  | Cis and trans |
|  |  |  |  | 35 Schizophrenia |  |  |  |  | 19-59  mean 42.6 | 74% male  26% female | 35 Caucasian |  |  |  |
|  |  |  |  | 35 Bipolar |  |  |  |  | 19-64  mean 45.3 | 49% male  51% female | 35 Caucasian  1 African American  1 Native American |  |  |  |
| [Liu *et al*. [28](#_ENREF_28)] | 127 | Prefrontal Cortex | 127 | 39 Bipolar  37 Schizophrenia  11 Depression  40 Control | 7 167 | | 5 286 | | 20-65  median 45 | 65% male  35% female | Caucasian | Affymetrix Human Genome U133A | Affymetrix GeneChip Mapping 5.0K | Cis and trans |
| [Myers *et al*. [31](#_ENREF_31)] | 193 | Cortex (Pooled data from 20% frontal, 70% temporal and 1% parietal) | 193 | Normal | 25 866 | | 3 709 | | 65-100  avg 81 | 54% male  46% female | Caucasian | Illumina Human  Refseq-8 | Affymetrix GeneChip Human Mapping 500K | Cis and trans |
| [Webster *et al*. [29](#_ENREF_29)] | 364 | Cortex (Pooled from 21% frontal, 73% temporal, 2% parietal and 3% cerebellar) | 188 | Normal | 2 485 | | 743 | | 65-100  avg 81 | 55% male  45% female | Caucasian | Illumina Human Refseq-8 | Affymetrix GeneChip Human Mapping 500K | Cis and trans |
|  |  | Cortex (Pooled from 18% frontal, 60% temporal, 10% parietal and 13% cerebellar) | 176 | Late-onset AD |  |  |  |  | 68-102  avg 84 | 50% male  50% female | Caucasian |  |  |  |
| [Zou *et al*. [30](#_ENREF_30)] | ~400 | Cerebellum | 197 | AD | 5 370 | | 625 | | Mean±-SD  73.6 ± 5.6 | 49% male  51% female | Caucasian | Illumina HumanHT-12 v4.0 | Illumina Human  Hap300-Duo | Cis only |
|  |  |  | 177 | Other pathologies |  |  |  |  | 71.7 ± 5.5 | 64% male  36% female | Caucasian |  |  |  |
|  |  | Temporal Cortex | 202 | AD |  |  |  |  | 73.6 ± 5.5 | 47% male  53% female | Caucasian |  |  |  |
|  |  |  | 197 | Other pathologies |  |  |  |  | 71.6 ± 5.6 | 60% male  40% female | Caucasian |  |  |  |
