# Supplementary material for: Overlap of expression Quantitative Trait Loci (eQTL) in human brain and blood
Source: BMC Med Genomics. 2014 Jun 3;7:31. doi: 10.1186/1755-8794-7-31 (PMC4066287; doi:10.1186/1755-8794-7-31)
Supplement: Additional file 2: Table S2 — Overlap of eQTL was evaluated using the SNP proxy lists generated under two linkage disequilibrium threshold (r 2 = 0.8 and r 2 = 0.5). [file 1755-8794-7-31-S2.docx]

**Supplementary table 2 |** Overlap of eQTL was evaluated using the SNP proxy lists generated under two linkage disequilibrium threshold (*r^2^*=0.8 and *r^2^*=0.5).

| Study | r^2^ > 0.8 | r^2^ > 0.5 |
| --- | --- | --- |
| Colantuoni *et al*. [17] | 12 | 12 |
| Gibbs *et al*. [12] | 34 | 34 |
| Heinzen *et al*. [7] | 0 | 0 |
| Kim *et al*. [18] | 8 | 9 |
| Liu *et al*. [19] | 7 | 7 |
| Myers *et al*. [22] | 49 | 50 |
| Webster *et al*. [20] | 17 | 17 |
| Zou *et al*. [21] | 16 | 16 |
