# Supplementary material for: Overlap of expression Quantitative Trait Loci (eQTL) in human brain and blood
Source: BMC Med Genomics. 2014 Jun 3;7:31. doi: 10.1186/1755-8794-7-31 (PMC4066287; doi:10.1186/1755-8794-7-31)
Supplement: Additional file 4: Table S3 — Genes with an overlapping eQTL reported in Westra et al. and brain studies. [file 1755-8794-7-31-S4.docx]

**Supplementary Table 3 |** Genes with an overlapping eQTL reported in Westra et al. and brain studies

| **Gene** | **Name** | **Number of brain studies reporting** |
| --- | --- | --- |
| NSFL1C | NSFL1 (p97) cofactor (p47) | 6 |
| PEX6 | peroxisomal biogenesis factor 6 | 6 |
| CDK5RAP2 | CDK5 regulatory subunit associated protein 2 | 5 |
| CDS2 | CDP-diacylglycerol synthase (phosphatidate cytidylyltransferase) 2 | 5 |
| CHURC1 | churchill domain containing 1 | 5 |
| CRIPT | cysteine-rich PDZ-binding protein | 5 |
| HMBOX1 | homeobox containing 1 | 5 |
| MRPL43 | mitochondrial ribosomal protein L43 | 5 |
| NAPRT1 | nicotinate phosphoribosyltransferase domain containing 1 | 5 |
| NSUN2 | NOP2/Sun RNA methyltransferase family, member 2 | 5 |
| RABEP1 | rabaptin, RAB GTPase binding effector protein 1 | 5 |
| ZNF266 | zinc finger protein 266 | 5 |
| ABHD12 | abhydrolase domain containing 12 | 5 |
| PILRB | paired immunoglobin-like type 2 receptor beta | 5 |
